# Random Amplified Polymorphic DNA Typing of Clinical and Environmental *Aeromonas hydrophila* Strains from Limpopo Province, South Africa

**DOI:** 10.3329/jhpn.v28i1.4517

**Published:** 2010-02

**Authors:** J.N. Ramalivhana, C.L. Obi, A. Samie, C. Labuschagne, G.F. Weldhagen

**Affiliations:** ^1^ College of Agriculture and Environmental Sciences, School of Agriculture and Life Sciences, University of South Africa, Pretoria, South Africa; ^2^ Academic and Research Division, Walter Sisulu University, Nelson Mandela Drive, Mthatha, Eastern Cape, South Africa; ^3^ Department of Microbiology, University of Venda, Private Bag X5050, Thohoyandou 0950, South Africa; ^4^ Inqaba Biotechnological Laboratory, Pretoria, South Africa; ^5^ Infectious Diseases Unit, Department of Internal Medicine, Faculty of Health Sciences, University of Pretoria and Molecular Biology Laboratory, AMPATH National Laboratory Service, Pretoria, South Africa

**Keywords:** *Aeromonas*, *Aeromonas hydrophila*, Diarrhoea, Genotyping, South Africa

## Abstract

The aim of the present study was to determine the genetic relatedness of strains isolated from diarrhoeal stool and water specimens collected from water-storage containers from different geographical areas in the Limpopo province. In total, 32 *Aeromonas* strains isolated from stool specimens collected from HIV/AIDS patients suffering from gastroenteritis and their household drinking-water stored in 20-L and 25-L containers were analyzed by random amplified polymorphic DNA PCR (RAPD). The RAPD fingerprints obtained proved reproducible when repeated on three different occasions using whole-cell DNA isolated from the *Aeromonas* strains. In total, 12 unique RAPD fingerprints were found. The results revealed a tendency of the isolates to cluster according to their origin of isolation (best-cut test 0.80 and bootstrap values >50%). However, a certain degree of similarity was also observed between isolates of water sources and clinical sources which indicated genetic relatedness. There were also genetic similarities between the clinical and the environmental strains of *Aeromonas* spp. isolated from different geographical areas. This study has demonstrated the genetic relatedness of *Aeromonas hydrophila* isolates from household drinking-water and clinical sources in South Africa, which may be due to cross-contamination from water to patients or vice-versa. This observation is of public-health significance, particularly in the era of HIV/AIDS. This study points to the importance of monitoring and evaluating infection-control measures for improved hygiene and to prevent cross-contaminations.

## INTRODUCTION

The genus *Aeromonas* comprises several species of oxidase-negative and catalase-positive, glucose-fermenting, facultative anaerobic, Gram-negative, rod-shaped, motile and non-motile bacteria (1). They are widely distributed in nature, especially in aquatic environments and have been isolated from various raw foods, such as fish and vegetables. These bacteria are widely found in surface water and sewage; they also occur in untreated and treated drinking-water (2). In humans, *Aeromonas* species are responsible for gastroenteritis, chronic diarrhoea, wound infections, respiratory tract infections, peritonitis, urinary tract infections, and septicaemia (3). *Aeromonas* spp. have been implicated as diarrhoea-causing agents in both HIV-positive and HIV-negative patients in South Africa (4). However, no study has determined the possible genetic relationship between environmental and clinical strains.

Genomic fingerprinting methods, such as random amplified polymorphic DNA PCR (RAPD) typing, are regarded as accurate methods for the typing of microorganisms for epidemiological purposes (5).

Although *Aeromonas* spp. have been described in South Africa, there is a paucity of studies that have determined the possible source of human infections, including the phylogenetic relatedness between clinical and environmental isolates. In the present study, RAPD was employed to determine the genetic relatedness of strains isolated from diarrhoeal stool and water specimens collected from water-storage containers from different geographical areas in the Limpopo province, South Africa.

## MATERIALS AND METHODS

### Study sites and ethical clearance

Stool samples and household drinking-water samples were collected from HIV and AIDS patients in different locations in the Limpopo province, including Belabela, Madombidzha, Mankweng (Polokwane), and Musina. *Aeromonas* spp. were isolated from these samples and characterized as previously described (6). Thirty-two strains of *Aeromonas hydrophila* from these samples were used in the present study for beta-lactamase production, antibiotic susceptibility as previously described (6), and in genotyping studies. The geographical origins and sources of the strains used in the study are indicated in the table. Ethical clearance was obtained from the Ethics and Research Committee of the University of Venda, Thohoyandou, Limpopo province, South Africa.

### Genomic DNA extraction

Extraction of whole-cell DNA was performed by a precipitation-based method as described previously (7). Briefly, the cells were lysed by the addition of SDS (Promega, Madison, WI) and lysozyme (Sigma Chemical Co. St. Louis, MO, USA) with incubation at 37 °C (QBT2 heating block, Grant Instruments Ltd., Cambridge, United Kingdom) for one hour. The DNA was further isolated using chloroform/isoamyalcohol (24:1) (Sigma Chemical Co., St. Louis, MO, USA) and precipitated with isopropanol (Merck, Darmstadt, Germany) at −20 °C overnight and stored at −20 °C until further analysis.

### Confirmation of *Aeromonas hydrophila* identity by 16S rRNA gene-sequencing

All the *Aeromonas* strains used in the present study were identified as previously described and confirmed by PCR analysis and sequencing of the 16S rRNA gene for two isolates. The 16S rRNA gene sequence was PCR-amplified in a Perkin-Elmer GeneAmp PCR System 2400 thermocycler as previously described (5)

All DNA extractions and PCR products were verified by gel electrophoresis for one hour in a 1% agarose gel (Pronadisa, Madrid, Spain) containing ethidium bromide (Promega, Madison, WI, USA) and the images captured using a digital gel documentation system (DigiDoc-It imaging system, UVP, Upland, CA, USA).

### Random amplified polymorphic DNA analysis of strains

Random amplified polymorphic DNA analysis was performed on 32 isolates using whole-cell DNA as template to determine the genetic relationships of isolates according to a modified method previously described (8, 9) using three different primers to obtain a banding pattern representative of the whole genome. All the samples were run at least twice using each primer.

### Analysis of RAPD fingerprints

The amplification products were electrophoresed in 1.5% agarose gel in Tris-borate buffer. Computer analyses were carried out using the GelCompar II software (version 3.0; Applied Maths, Kortrijk, Belgium). Similarity between fingerprints was calculated with the Dice coefficient. Cluster analysis was performed using the unweighted pair-group method with average linkages (UPGMA).

## RESULTS

The fingerprints of the isolates generated between 2 and 17 bands ranging from 100 to 3,500 bp. The banding patterns proved reproducible when repeated on two separate occasions. Random amplified polymorphic DNA fingerprints which possessed more than 90% similarity were considered identical and were assigned an RAPD type. On this basis, 12 unique RAPD types were assigned as shown in the figure. The isolates and the specimen types from which the isolates were obtained are shown in the table.

There was a difference between the clinical and the environmental strains. Type 1, for example, was composed of four clinical strains, and type 4 was composed of five clinical strains while type 7 was composed of all four environmental strains. However, there was a mixture of clinical and environmental strains in the same type.

## DISCUSSION

We had previously isolated and characterized *Aeromonas* spp. from stool and water samples from different areas in the Limpopo province (6). However, previous studies were not able to fully ascribe any genetic relatedness between the environmental and the clinical isolates. In the present study, three primers were used for RAPD fingerprinting of 32 *A. hydrophila* strains from stools and water samples from HIV and AIDS patients from five different localities approximately 400 km apart throughout the Limpopo province situated in the northern part of South Africa.

**Table:** Characteristics and random amplified polymorphic types of *Aeromonas hydrophila* strains used in the present study

Locality	Isolate*	RAPD type	Beta-lactamase production	Antibiotic resistance combination
Belabela	6165S	1	Negative	Ampicillin
Belabela	6168S	1	Negative	Ampicillin
Belabela	6173S	4	Negative	Amikacin, ampicillin, cefotaxime, nitrofurantoin, tobramycin
Belabela	6197S	4	Positive	Amikacin, ampicillin, cefazolin, cefotaxime
Belabela	6237S	5	Positive	Ampicillin, cefotaxime, nitrofurantoin, tobramycin
Belabela	6164E	5	Negative	Ceftriaxone, ciprofloxacin, gentamicin, ampicillin
Belabela	6249E	6	Positive	Amikacin, ampicillin, cefazolin, cefotaxime, nitrofurantoin, tobramycin
Belabela	6241E	7	Negative	Nitrofurantoin, amikacin, ampicillin
Belabela	6238S	12	Negative	Nitrofurantoin
Belabela	6248E	11	Negative	Ampicillin
Madombidhza	6171S	1	Negative	Ampicillin
Madombidhza	6170S	4	Negative	Ampicillin, nitrofurantoin, tobramycin
Madombidhza	6199S	6	Positive	Ampicillin, cefazolin, nitrofurantoin, tobramycin
Madombidhza	6250E	7	Negative	Ampicillin
Madombidhza	6183S	10	Negative	Ampicillin, cefazolin, ampicillin
Madombidhza	6184S	11	Negative	Ampicillin
Mankweng	6242E	8	Negative	Amikacin, ampicillin, cefazolin, cefotaxime, nitrofurantoin, tobramycin
Mankweng	6258E	7	Negative	Ampicillin
Mankweng	6166S	1	Negative	Ampicillin
Mankweng	6189E	6	Negative	Tobramycin
Mankweng	6182S	5	Negative	Ampicillin
Mankweng	6255E	11	Negative	Nitrofurantoin, tobramycin
Mankweng	6172S	11	Negative	Amikacin, ampicillin, cefazolin, cefotaxime, nitrofurantoin, tobramycin
Musina	6257E	2	Negative	Nitrofurantoin
Musina	6175S	4	Negative	Ampicillin, cefazolin
Musina	6174S	3	Negative	Amikacin, ampicillin, cefazolin, cefotaxime, nitrofurantoin, tobramycin
Musina	6239E	5	Negative	Ampicillin
Musina	6192E	5	Negative	Ampicillin
Musina	6190E	6	Negative	Ampicillin
Musina	6240E	7	Negative	Ampicillin
Musina	6247E	9	Negative	Tobramycin
Musina	6198S	4	Negative	Amikacin, ampicillin, cefazolin, cefotaxime, nitrofurantoin, tobramycin

*At the end of the code: E=Environmental (water samples);

S=Stool

The banding patterns obtained in the present study were similar to those obtained by other researchers (5).

The results indicate a high genetic diversity in the *A. hydrophila* population in South Africa even though there was a mixture of environmental and clinical strains in some RAPD types probably due to separate introductions of the strains in the localities and poor health practices (10, 11). Other studies around the world, using genetic methods, have described high diversity in the *A. hydrophila* population (12, 13). Previous studies have indicated that homologs of a gene encoding a protein with lipase activity appeared to be widely distributed in *Aeromonas* strains, probably associating with the evolutionary genetic difference between clinical and environmental isolates of *A. hydrophila* (14). We have previously shown that clinical strains of *A. hydrophila* possessed pathogenic characteristics, such as haemolytic and haemagglutinating activities (6). It is, thus, possible that the environmental strains that had similar RAPD profiles as clinical strains might share pathogenic characteristics as well. Studies of *A. hydrophila* in Japan, using randomly-amplified polymorphic DNA-PCR (RAPD-PCR), revealed one specific RAPD pattern group (G) that was associated only with strains from environmental sources. The comparison of isolates with pattern group G with a set of isolates derived from human blood showed low induction of cytotoxicity from those with RAPD pattern group G suggesting low virulence of these strains (13).

Although antibiotic resistance was common, statistical analysis using the chi-square test indicated that RAPD type 4 was more associated with amikacin resistance (χ^2^=3.872; p=0.049) and resistance to cefotaxime (χ^2^=3.872; p=0.049). Of all the strains tested, four were beta-lactamase-positive, of which two were of RAPD type 6. Statistical analysis indicated that RAPD type 6 was more associated with beta-lactamase production (χ^2^=5.878, p=0.015). Multiple drug resistance (MDR) defined as resistance of a specific strain to more than two antibiotics was common (41%) among the strains tested. Statistical analysis indicated that RAPD type 4 was more associated with MDR (χ^2^=4.567, p=0.033). In a previous study, there was a correlation between the antibiotic resistance profile and the RAPD profiles of specific *Aeromonas* isolates (15).

In the present study, we did not find a clear differentiation between strains from different localities. RAPD type 1 was not found in Musina area but was found in all the other areas, including Belabela, Mankweng, and Madombidzha. Some RAPD genotypes included only one strain (Fig.). For example, RAPD genotype 2, type 3, and type 9 were all obtained from Musina area and included the environmental and clinical isolates while type 8, 10, and 12 were obtained from Mankweng, Madombidzha, and Belabela respectively. This diversity indicates the need for regular monitoring of *Aeromonas* strains for pathogenic characteristics and for the possibility of epidemic strains that could be circulating in the population. Studies of clinical and environmental ecotypes of *Aeromonas* in Mexico demonstrated the high intra-specific diversity within *A. hydrophila* and revealed a clear differentiation of strains according to their ecological origins (16). The same study also confirmed, based on the distribution of virulence-related genes, that *A. hydrophila* is a genetically-heterogeneous species that harbour ecotypes which have different pathogenic potentials to humans and other animals.

**Fig.**
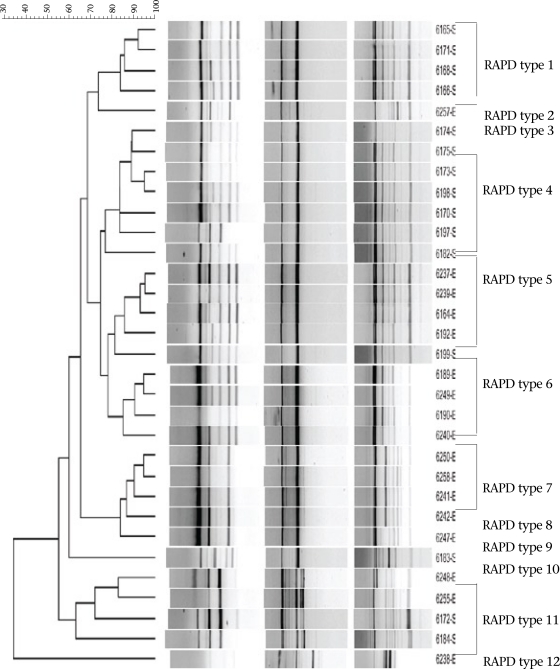
Dendogram obtained through RAPD typing with primer 277, H1, and 275 depicting the relationship between *Aeromonams hydrophila* stool isolates and water isolates obtained from Limpopo

RAPD=Random amplified polymorphic DNA PCR

The results indicating the genetic similarity between the clinical and the environmental isolates of *A. hydrophila* in this study do not accord with those obtained in Switzerland that showed no genetic relationship between clinical and environmental strains (17). Studies in France also found that the water sampled in the hospital was not the source of infections for patients at the University Hospital in Marseille (18). With the high prevalence of HIV and AIDS in South Africa, patients might become more susceptible to infection by *Aeromonas,* and the possibility of acquiring the organisms from water is high and represents a great threat to human health. The results of the present study have revealed the possibility of an intricate web of relationship between isolates from stool and water samples in the Limpopo province of South Africa, indicating cross-contaminations and points to the need for effective control measures. The present study also showed that specific genotypes might be related to antibiotic resistance. Further studies on the cytotoxicity of strains under investigation are warranted and will further clarify the pathogenic characteristics of *A. hydrophila* strains in the region.

## ACKNOWLEDGEMENTS

The authors are grateful to Dr. Jane Wright and Dr. Oliver Preisig of Inqaba Biotech Laboratory, Pretoria, South Africa, for their various roles in the implementation of the project. They are also immensely grateful to the National Research Foundation, South Africa, for financial assistance.

## References

[1] Howard SP, Macintyre S, Buckley JT, Austin B, Altwegg M, Gosling PJ, Joseph S (1996). Toxin. The genus Aeromonas.

[2] Maalej S, Mahjoubi A, Elazri C, Dukan S (2003). Simultaneous effects of environmental factors on motile *Aeromonas* dynamics in an urban effluent and in the natural seawater. Water Res.

[3] Martínez JA, Pozo L, Almela M, Marco F, Soriano A, López F (2007). Microbial and clinical determinants of time-to-positivity in patients with bacteraemia. Clin Microbiol Infect.

[4] Obi CL, Bessong PO (2002). Diarrhoeagenic bacterial pathogens in HIV-positive patients with diarrhoea in rural communities of Limpopo province, South Africa. J Health Popul Nutr.

[5] Alavandi SV, Ananthan S, Pramod NP (2001). Typing of *Aeromonas* isolates from children with diarrhoea & water samples by randomly amplified polymorphic DNA polymerase chain reaction & whole cell protein fingerprinting. Indian J Med Res.

[6] Obi CL, Ramalivhana J, Samie A, Igumbor EO (2007). Prevalence, pathogenesis, and antibiotic susceptibility profiles of *Aeromonas* isolates from stool samples of patients in the Venda region of South Africa. J Health Popul Nutr.

[7] Balcázar JL, Vendrell D, de Blas I, Ruiz-Zarzuela I, Gironés O, Múzquiz JL (2007). Quantitative detection of *Aeromonas salmonicida* in fish tissue by real-time PCR using self-quenched, fluorogenic primers. J Med Microbiol.

[8] Campbell M, Mahenthiralingam E, Speert DP (2000). Evaluation of random amplified polymorphic DNA typing of Pseudomonas aeruginosa. J Clin Microbiol.

[9] Zemanová E, Jirků M, Mauricio IL, Miles MA, Lukes J (2004). Genetic polymorphism within the *leishmania donovani* complex: correlation with geographic origin. Am J Trop Med Hyg.

[10] Andargie G, Kassu A, Moges F, Tiruneh M, Huruy K (2008). Prevalence of bacteria and intestinal parasites among food-handlers in Gondar town, northwest Ethiopia. J Health Popul Nutr.

[11] Eshcol J, Mahapatra P, Keshapagu S (2009). Is fecal contamination of drinking water after collection associated with household water handling and hygiene practices? A study of urban slum households in Hyderabad, India. J Water Health.

[12] Korbsrisate S, Trakulsomboon S, Damnin S, Gatedee J, Ratchtrachenchai OA, Leelaporn A (2007). Genetic variations in Aeromonas hydrophila isolates from clinical and environmental sources in Thailand. Southeast Asian J Trop Med Public Health.

[13] Lee MF, Peng CF, Lin YH, Lin SR, Chen YH (2008). Molecular diversity of class 1 integrons in human isolates of *Aeromonas* spp. from southern Taiwan. Jpn J Infect Dis.

[14] Watanabe N, Morita K, Furukawa T, Manzoku T, Endo E, Kanamori M (2004). Sequence analysis of amplified DNA fragments containing the region encoding the putative lipase substrate-binding domain and genotyping of *Aeromonas hydrophila*. Appl Environ Microbiol.

[15] Gunsalam JW, Radu S, Benjamin PG, Selamat J, Robin T (2006). Evidence of cross-contamination of *Aeromonas hydrophila* by fingerprinting: significance for food safety. J Food Safety.

[16] Aguilera-Arreola MG, Hernández-Rodríguez C, Zúñiga G, Figueras MJ, Castro-Escarpulli G (2005). *Aeromonas hydrophila* clinical and environmental ecotypes as revealed by genetic diversity and virulence genes. FEMS Microbiol Lett.

[17] Moyer NP, Luccini GM, Holcomb LA, Hall NH, Altwegg M (1992). Application of ribotyping for differentiating aeromonads isolated from clinical and environmental sources. Appl Environ Microbiol.

[18] Davin-Regli A, Bollet C, Chamorey E, Colonna D'istria V, Cremieux A (1998). A cluster of cases of infections due to *Aeromonas hydrophila* revealed by combined RAPD and ERIC-PCR. J Med Microbiol.

